# Influence of Whitening Gel Application Protocol on Dental Color Change

**DOI:** 10.1155/2015/420723

**Published:** 2015-03-17

**Authors:** Taciana Marco Ferraz Caneppele, Carlos Rocha Gomes Torres, Maria Filomena Rocha Lima Huhtala, Eduardo Bresciani

**Affiliations:** Department of Restorative Dentistry, Institute of Science and Technology, Universidade Estadual Paulista (UNESP), Avenue Francisco José Longo 777, 12245-000 São José dos Campos, SP, Brazil

## Abstract

*Objectives.* To evaluate the influence of different whitening protocols on the efficacy of 35% hydrogen peroxide (HP) tooth whitening and gel pH and concentration.* Material and Methods.* Eighty-four enamel/dentin discs from bovine incisors were used. The baseline color was measured with a spectrophotometer. Two sessions of in-office whitening with 35% HP were performed under different protocols: G1: 3 applications of HP (10 min each) per session; G2: 1 application of 30 min per session; G3: 1 application of 40 min per session, with no gel replenishment within session for groups 2 and 3. HP titration and pH evaluation at baseline, after 10, 30, and 40 min were also performed. The final color was measured 24 h after the 1st and 2nd whitening sessions. Data were submitted to Repeated Measures ANOVA and Tukey's test.* Results.* For color evaluation, no differences were observed among groups after two sessions. HP titration showed no drop on concentration after 10, 30, or 40 min. The pH was 5.54 at baseline and 5.41 after 40 min.* Conclusion.* Replenishment or extended application time of in-office whitening gel does not affect gel pH and concentration, a fact that supports the similar effectiveness of whitening observed among the tested protocols.

## 1. Introduction

Tooth whitening offers a conservative, simplified, and economical alternative to modify the color of teeth. Currently, three main approaches are adopted for vital tooth whitening, which are at-home, in-office, and over-the-counter whitening therapies [1].

Several studies have been conducted to evaluate the efficacy of different products and protocols [2–4] for tooth whitening, and the results are usually conflicting. Three factors present noticeable influence on the activity of whitening agents, which are often involved in the kinetics of most chemical reactions. They are heat, concentration, and pH [5]. Regarding heating, all whitening agents act faster with increasing temperature. Heat accelerates the permeation of hydrogen peroxide into enamel and its action on stained substance [6]. However, heat may be hazardous to pulp cells, once it might aggravate pulp damage in contact to hydrogen peroxide [7]. Regarding gel concentration, the presence of greater number of reactive molecules generally leads to increased activity, although the response performance at higher concentrations is not necessarily linear for whitening products. With respect to pH, alkalinity accelerates the decomposition of hydrogen peroxide. Thus, some whitening products are presented in two bottles, one with hydrogen peroxide solution in acidic pH (stable) and another with alkaline pH. When solutions are mixed, the pH becomes neutral, resulting in degradation/activity of hydrogen peroxide. Over time, with the degradation of hydrogen peroxide, the pH would become acidic, decreasing the effectiveness of peroxide action and possibly leading to damage to tooth structure. This last finding would support the rationale for replenishing the whitening gel during in-office whitening therapies.

According to some manufacturers' instructions, during in-office whitening, highly concentrated hydrogen peroxide whitening gel is left on tooth surface for 5 to 20 minutes and replenished 2 or 3 times. However, a certain amount of time is necessary for the hydrogen peroxide to act on enamel [8] and the decomposition of hydrogen peroxide requires a certain period of time when no activator is used.

However, the effect of different in-office whitening protocols on the pH and concentration of the gel during the treatment has not been investigated. Thus, the aim of this study was to evaluate the gel pH and concentration, and the efficacy of 35% hydrogen peroxide (HP) tooth whitening with different application times using enamel/dentin substrate.

## 2. Material and Methods

### 2.1. Color Measurement

The specimens were prepared according to the method described by Wiegand et al. [9]. Eighty-four extracted, nondamaged, and intact bovine incisors were used. The teeth were obtained from a local slaughterhouse. Enamel-dentin specimens presenting 3 mm in diameter and 2 mm in height (1 mm of enamel and 1 mm of dentin) were prepared from the buccal surface using a trephine mill. Prior to treatment, the baseline color of each specimen was assessed under standardized ambient conditions according to the CIE *L* ^*^ *a* ^*^ *b* ^*^ system, using a spectrophotometer (CM2600d, Konica Minolta). The device was adjusted to use the D65 standard light source with 100% UV included or 100% UV excluded and specular reflection included (SCI). The observer angle was set at 2° and the device was adjusted to a small reading area (SAV). The color of each sample was assessed 3 times and averaged. The results of color measurements were quantified in terms of three coordinate values (*L* ^*^, *a* ^*^, *b* ^*^), as established by the Commission Internationale de l'Eclariage (CIE), which locates the color of an object in a three-dimensional color space. Axis *L* ^*^ represents the degree of lightness within a sample and ranges from 0 (black) to 100 (white). Axis *a* ^*^ represents the degree of green/red color, while *b* ^*^ axis represents the degree of blue/yellow color within the sample. The *L* ^*^ value of each specimen was used for stratified allocation among 3 groups. The color was measured over white (*L*: 84.95; *a*: −0.38; *b*: 2.93) standard backgrounds. From the color measurement at baseline and those after the whitening procedures, the values of the changes of *L* ^*^ (Δ*L*), *a* ^*^ (Δ*a*), and *b* ^*^ (Δ*b*) were calculated. Next, the total change color or the variation in perception of color of each specimen was calculated, designated by the abbreviation Δ*Eab*. This parameter was calculated according to the following formula:

(1) $$\begin{array}{c} \Delta E*ab={\left(\Delta L^{2}+\Delta a^{2}+\Delta b^{2}\right)}^{1/2}. \end{array}$$

### 2.2. Whitening Procedure

An experimental 35% HP gel, composed of two components, was used. The first component was a solution of 50% hydrogen peroxide containing an acrylic thickener, which is a white solution in an acidic environment (solution A—pH 1.5). The second component was an aqueous solution containing an alkaline substance (solution B—pH 11.3). In order to produce the final whitening gel (pH 6.5) three parts of solution A and one part of solution B, by volume, were mixed.

Once mixed, a 2 mm thick layer (approximately 0.1 g) of the whitening gel was applied over the surface of the specimens.

Three different protocols of application were performed.

Group 1, 3 × 10: the gel was allowed to remain on specimens' surface for 10 min, with gentle stirring after 5 min using a plastic instrument to dislodge the bubbles formed. After ten minutes, the gel was removed using a vacuum aspirator and two additional applications were performed, for a total of 30 min of active whitening.

Group 2, 1 × 30: the gel was allowed to remain on specimens' surface for 30 min, with gentle stirring each 5 min using a plastic instrument to dislodge the bubbles formed.

Group 3, 1 × 40: the gel was allowed to remain on specimens' surface for 40 min, with gentle stirring each 5 min using a plastic instrument to dislodge the bubbles formed.

All specimens were stored in artificial saliva for 7 days and the whitening procedure was repeated. The color measurements were performed after the specimens were stored in artificial saliva for 24 h (after first and second whitening sessions).

### 2.3. pH Evaluation

Gel pH was measured using a portable pH meter (Seven Multi, Mettler Toledo Inc., Canton, MA, USA) with a direct electrode, which was calibrated with standard buffer solutions at pH 4.0 and 6.86 prior to analysis. The pH was assessed at baseline and after 10, 30, and 40 min. Additional five teeth were used, at each the periods described above, to allow the collection of certain volume of hydrogen peroxide for pH measurement. Five measurements per period were performed and the mean was calculated.

### 2.4. HP Titration

The titration method, using 0.1 N potassium permanganate solution, assessed the correct concentration of H_2_O_2_ from the pure 50 w% H_2_O_2_ solution (Sigma–Aldrich, Buchs, Switzerland) used to prepare the gel, and also assessed HP concentration during the whitening process. Primary standard sodium oxalate was used to standardize the 0.1 N KMnO_4_ solution [10].

A 2 mm layer of whitening gel was applied over five additional bovine incisors and collected after 10, 30, and 40 min of service, for titration analysis. Five titrations for each evaluated time were performed and the HP mean concentration was calculated.

**Figure 1:**
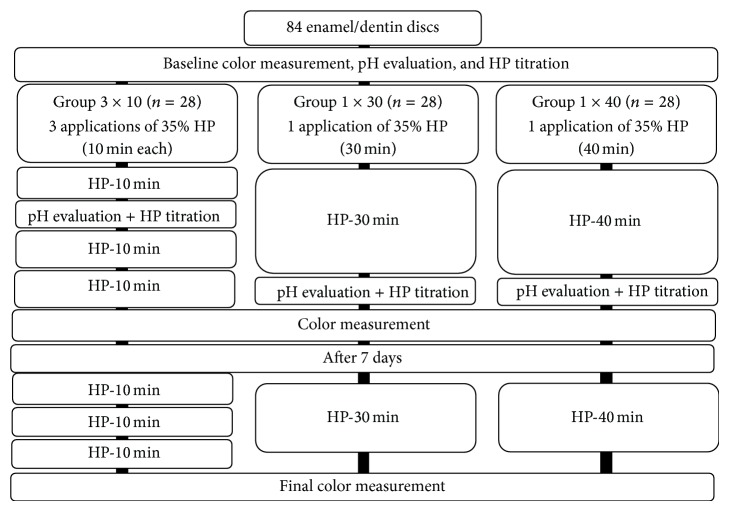
Schematic representation of the design of the study.

Figure 1 presents the study design.

### 2.5. Statistical Analysis

Obtained data were statistically analyzed using Statistica for Windows (Statsoft). Repeated measures analysis of variance and Tukey's test were applied at a significant level of 0.05 for color, pH, and concentration evaluation.

## 3. Results

### 3.1. Whitening Efficacy

The overall color coordinates measurements are shown in Table 1.

For color changes, based on repeated measures ANOVA, there were significant differences between the whitening sessions (*P* < 0.05), but no differences were found between the different protocols (*P* > 0.05).

**Figure 2:**
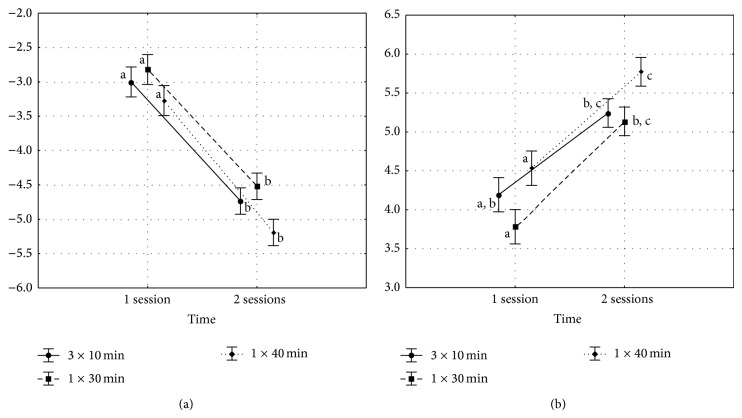
Results of Δ*b* (a) and Δ*Eab* (b). Vertical bars denote ± standard errors, and sets with the same letters are not significantly different (Tukey's Test, *P* > 0.05).

Figure 2 shows the results of Δ*b* and Δ*Eab*. After two sessions, for all experimental conditions there was an increase of color change.

### 3.2. HP Concentration and pH Evaluation

**Figure 3:**
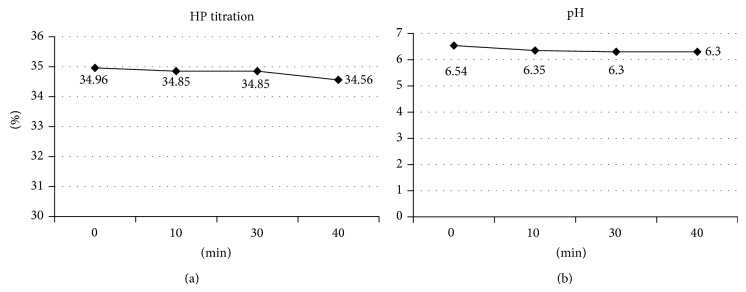
HP titration (a) and pH evaluation (b).

For HP titration, no statistical difference was observed at baseline and during the periods of evaluation (*P* > 0.05). Also for pH evaluation, although a small drop on pH was observed during the treatment time, it was not statistically significant (*P* > 0.05).  Figure 3 shows the results for both analyses.

## 4. Discussion

This study compared different protocols of in-office whitening regarding efficacy (color change), pH, and concentration of HP.

Previous studies have evaluated the outcomes of tooth whitening using the Δ*Eab* formula. However, there is no consensus on which of the parameters (Δ*L*, Δ*a*, Δ*b*, or Δ*Eab*) would be best suitable to predict adequate whitening results. Li [11] reported that the most critical challenge in the evaluation of color with spectrophotometers and colorimeters is the lack of methods for interpreting data from instruments in relation to clinical changes of dental color. Bengel [12] observed that major changes occur in the values of *L* ^*^ and *b* ^*^ coordinates after whitening, and the coordinate *b* ^*^ would be more relevant in assessing the whitening treatment. For that author, Δ*Eab* does not reflect the total color change. Karpinia et al. [13] observed a significant reduction of yellowing (Δ*b*) and an increased lightness (Δ*L*) after whitening procedures. In the present study, the greatest differences before and after whitening were observed for the *b* ^*^ coordinate (Table 1, Figure 2(a)).

For all groups, after the second session, there was an increase on the degree of whitening and it may be observed by the increase of Δ*b* and Δ*Eab* (Table 1 and Figure 2).

Tooth enamel is the densest tissue in the human body and has a very low permeability. Therefore, the permeation of hydrogen peroxide is slow [14]. Because a certain amount of time is necessary for the hydrogen peroxide to act on enamel [8], and as the decomposition of hydrogen peroxide takes place a long period of the time, it was tested the prolonged application time of peroxide without replenishing of the gel. The results of this study showed that replenishing the gel every 10 min for 3 times per section (as recommended by some manufacturers) did not affect the efficacy of treatment. In agreement with this research, Al-Harbi et al. [15] also observed no significant difference in the effectiveness of treatment when performed 2 applications of 30 min or 4 of 15 min. Similar results were obtained in an in vitro study carried by Kwon et al. [16]. The authors also found that there was significantly greater hydrogen peroxide penetration in the pulp when the gel was replenished during the treatment. However, Reis et al. [17] observed an increase of tooth sensitivity levels when prolonged application time of hydrogen peroxide was performed.

The extended time of 40 min was not able to significantly increase the whitening efficacy. Further studies should be carried to verify from that point the increase of application time could affect the efficacy of the treatment.

The present whitening efficacy behavior might be associated to no changes on gel pH and concentration at different assessed periods.

After forty minutes of gel application a small decrease in HP concentration on tooth surface was detected (not significant). This result indicates that a small amount of peroxide was degraded. Free radicals derived from the HP decomposition are slowly formed without the presence of a potent catalyst. In the present study, an increase in pH acts as catalyst and was obtained by mixing the 2 solutions immediately prior to use. Fornaini et al. [18] also observed a small decrease in the HP concentration in whitening gel, after 20 minutes in contact with the tooth structure. Thus, the rationale of replenishment of gel every 10 min because of the rapid degradation of hydrogen peroxide is not valid.

One concern with the whitening treatment is the possibility of demineralization of tooth enamel. Some whitening gels have acidic pH, which may favor this demineralization [19]. The neutral or basic pH of whitening agents might minimize changes in tooth structure and side effects such as tooth sensitivity and gingival irritation [20]. Also, according to Coons [5], alkaline pH accelerates the degradation of hydrogen peroxide by increasing the release of free radicals. Over time, with the degradation of hydrogen peroxide, the pH would become acidic, decreasing the efficacy of peroxide and also damaging the tooth structure. However, this study found that after 40 min a small, nonsignificant decrease in pH was observed, remaining at acceptable levels that are incapable of promoting enamel demineralization, and also reducing the degradation of the product.

## 5. Conclusion

Within the limitations of the present study it could be concluded that replenishment or extended time of application of in-office whitening gel does not affect the efficacy of the treatment and gel pH and concentration did not change during the 40 min of evaluation. All protocols were similar after one or two in-office sessions.

## Figures and Tables

**Table 1:** Mean and standard deviations of color parameters for all experimental conditions.

	Δ*L*		Δ*a*		Δ*b*		Δ*E*	
	1 session	2 sessions	1 session	2 sessions	1 session	2 sessions	1 session	2 sessions
3 × 10 min	−0.60 ± 2.74	−0.50 ± 2.14	0.96 ± 0.53	0.48 ± 0.36	−3.00 ± 1.15	−4.73 ± 1.02	4.19 ± 1.24	5.24 ± 1.00
1 × 30 min	−1.07 ± 1.88	−1.30 ± 1.86	1.17 ± 0.56	0.81 ± 0.49	−2.82 ± 1.03	−4.52 ± 0.86	3.78 ± 0.96	5.14 ± 0.82
1 × 40 min	−1.11 ± 2.55	−1.11 ± 2.06	1.41 ± 0.62	0.70 ± 0.65	−3.27 ± 1.26	−5.19 ± 1.15	4.53 ± 1.27	5.77 ± 1.08
